# Research on Shelf-Life Extension Technologies for Food Sustainability: An Assessment of Scientific Activities and Networks

**DOI:** 10.1155/2022/7120662

**Published:** 2022-08-12

**Authors:** Jakkrit Thavorn, Veera Muangsin, Chupun Gowanit, Nongnuj Muangsin

**Affiliations:** ^1^Technopreneurship and Innovation Management Program, Graduate School, Chulalongkorn University, Bangkok 10330, Thailand; ^2^Department of Computer Engineering, Faculty of Engineering, Chulalongkorn University, Bangkok 10330, Thailand; ^3^Department of Chemistry, Faculty of Science, Chulalongkorn University, Bangkok 10330, Thailand

## Abstract

A clearer understanding of research streams and players involved in efforts to address the sustainability of global food and agricultural systems is needed to clarify the current state of scientific knowledge and form collaborations to pursue future research directions. This study presents new insights into this issue through a scientometric process involving a case study of technologies for extending fruit shelf-life. The text mining software was utilized to analyze 3,131 Web of Science-indexed articles published between 2000 and 2020 as a means to glean the conceptual structure of current knowledge and conduct a social network analysis to explore scientific and publication activity. The findings were mapped onto a strategic diagram of research productivity and collaboration between players at the national, organizational, and individual levels. This research's main findings highlight that research on shelf-life technology is in continuous development, and academic institutions from China, Spain, and the U.S. are the core national players in this field. The results provide insights for further investigation to strengthen co-research and technological development programs in other fields. Researchers who are exploring networking opportunities can use the model and process presented as a guideline for identifying emerging and future research trends and formulating strategies.

## 1. Introduction

The 17 Sustainable Development Goals (SDGs) were developed by the United Nations Development Program (UNDP) and ratified by 193 countries. The SDGs aim to eliminate issues such as poverty and insecurity, with multiple SDGs tackling food-related issues [1]. The “zero hunger” pledge focuses on food security and sustainable agriculture, while “responsible consumption and production” seeks to address food losses. The “climate action” pledge includes efforts to cut food waste emissions, and “partnerships for the goals” prioritizes collaborative research to achieve these ambitions.

Recent years have witnessed emerging threats to global food safety and security together with the sustainability of food production systems. The world's population is expected to reach nearly 10 billion by 2050, and conservative projections of agricultural demand suggest a 50% increase compared with 2013 levels [2]. Agriculture accounts for approximately 4% of global gross domestic product (GDP), and in some developing countries, it contributes over 25% of GDP [3]. However, agriculture generates unsustainable levels of pollution and waste, accounting for 24% of global greenhouse gas emissions (GHG)—compared with 21% for the industrial sector. The mitigation efforts are critical to reducing its role in contributing to climate change [4]. In turn, climate change poses grave risks to food quality and nutrition, thereby representing a threat to agriculture-driven growth and efforts to achieve poverty reduction, and enhance food security and safety.

Food loss and food waste are major factors contributing to agricultural unsustainability. One-third of the world's food products are lost due to rotting or discarding, and actively confronting this problem is critical to addressing food and nutrition security and reducing environmental stress. More than 80% of losses and waste occurs due to postharvest management practices, namely, processing, packaging, distribution, and consumption [5]. Food loss and waste can worsen food quality and reduce food security, thereby increasing the risk of undernutrition [4].

Technologies for improving or prolonging foods' shelf-life can help deliver more quality food, thereby reducing food waste. Specifically, widespread demand for fresh produce has led to a global trend of growing consumption in recent years [2]. However, large proportions of fruits and vegetables never make it to market due to various processing operations that shorten shelf-life [6]. Increasing global demand for fresh fruit and vegetables over recent years has resulted in greater R&D investment. The high nutritional properties of these foods are believed to have driven this consumption pattern.

In light of the above, effective technologies to combat food loss are urgently needed to promote greater food safety, enhanced consumer protection, and the reduction or elimination of food waste. Research on ways to enhance shelf-life has substantially increased over the last two decades, and several bibliometric analyses have been conducted [7]. For example, Tatry et al. [7] analyzed research on fruit and vegetable species to identify leading actors and top species and topics to obtain overall productivity of the research landscape. However, most analyses focus on the food supply chain and security without considering the technologies being developed to prolong shelf-life. The comprehensive knowledge of the research streams and players involved in these efforts is also rarely observed.

Improving our understanding of the productivity and dynamics of collaborative relationships in science and technology can aid the formulation of strategies for future R&D efforts [8, 9]. Tracking complex R&D communities can be challenging due to their intricate multidisciplinary linkages involving various researchers and institutions across the sciences, engineering, humanities, and/or social sciences [10]. However, a number of researchers have developed tools and techniques to map R&D networks and forecast both emerging areas of investigation and future innovation pathways [11].

To address the abovementioned gaps, this article presents a scientometric analysis of the current state of research on technologies to extend the shelf-life as well as the dynamics and productivity of players using various scientometric indicators. We employed a strategic technology intelligence model that combines quantitative analysis with qualitative tools [12]. The technology expert involves in interpretations to elucidate techniques for clarifying and mapping the structure, patterns, and foci of collaborative relationships at multiple scales. The analysis of the conceptual structure knowledge and the social structure knowledge was combined to gain insights from technological points of view together with the network. In particular, we conducted a three-level analysis of collaborations at the national, institutional, and individual levels as well as a classification of associated research foci. Through an analysis of prominent keywords and clusters, we present publishing patterns (e.g., contributing countries, organizations, authors, and their relationships) and highlight the flows and evolution of major research themes and topics as well as overall research productivity in the area of this technology. Thereby, we present novel insights that contribute to a clearer understanding of the evolution, current state, and emerging trends of food shelf-life extension technology research.

In accordance with various factors such as disruptive technologies, speed of scientific publication, advanced technology of sciences, the industrial revolution, and the BCG economy, the following research questions are hypothesized:

1. What are the trends of this technology including the themes and subthemes that may be emerging, maturing, or declining?
2. Who are the key players at the country, organization, and researcher levels, how are they related, and what are the dynamics of the research collaborations among players?

To the best of our knowledge, this is the largest-scale scientometric study of publications concerning technologies to extend shelf-life. Not only an understanding of contributions emerges to this field but also various assessments of scientometric indicators assist in interpretation of scientific activities. For managerial contribution, this study could contribute to R&D's ability to identify specific technological trends and present a research-mapping proposal. These outputs help both practitioners explore professional communities and potentially form networks for future collaboration. An understanding of the substantial features of such networks could promote innovative and sustainable pathways to accelerate the R&D process, enhance competitiveness, and drive economic growth while ultimately generating global societal benefits [8].

## 2. A Scientometric Analysis in Fresh Produce Shelf-Life Extension Technologies

Scientometrics is an approach to measuring research productivity through the analysis of scientific documents. This analysis assists in revealing the emergence and development of research interests, and geographic and organizational distributions [13]. Scientometrics abounds in an array of fields such as environmental science [14], sustainability studies [15], and digitalization [16]. Scientometric analysis is commonly used to investigate database-indexed works to accomplish tasks ranging from journal impact measurements to the glean patterns of social, scientific, and technological progress [17]. Hence, the insightful knowledge is constructed to use as an aiding tool for managerial applications and forecasting future research and technological trends.

Food loss and waste are one of critical issues that need to be addressed. Technologies for improving or extending foods' shelf-life can help to solve this issue. Most research studies in scientometric analysis in this area mainly focuses on general scopes such as food chemistry [18], food security [19], food policy [7], and food supply chain [20]. Tatry et al. [7] used scientific articles to analyze leading actors, who contributed to most publications for food research. They mapped various types of food with countries and explored country-level collaboration. Xie et al. [19] studied the research status in food security in terms of policy and trends. They found that food supply, food use, and food access are the three pillars for future research direction.

In addition to the traditional food area, Bertoglio et al. [21] explored the evolution of digital technologies in agriculture. Main emerging research streams such as remote sensing, climate-smart agriculture, and artificial intelligence were revealed to adapt to reducing food loss and waste. Priyadarshi et al. [22] carried out risks in postharvest management, that is, one of the food supply chains that contribute to most food loss and waste. They analyzed keywords to provide a guideline for entrepreneurs.

Regarding scientometrics in terms of science and technology in this area, few studies were observed. For instance, Salgado–Cruz et al. [23] explored the use of the biopolymer material, called chitosan, in postharvest products. The bacteria and fungi affecting the quality of fruits and vegetables were investigated. Moreover, the coating materials with chitosan were revealed for the extension of fruits' shelf-life. This study showed deep analysis in terms of a technical point of view; however, there is a lack of social structure for R&D collaboration.

Therefore, scientometric analysis related to the technology of shelf-life has been rarely observed. As a consequence, our study proposed the scientometric technique to explore research areas in this particular field to fulfill gaps in terms of science and technology. In addition, we combined the conceptual structure analysis with a three-level social structure analysis. Thus, the findings are able to be used for the formulation of an R&D roadmap in the future.

## 3. Methodology and Data

We utilized scientometric text mining to identify potential technology opportunities together with research activities. The shelf-life extension technology was used as one of the cases to understand its development to deal with food loss and waste issues. This enables the tracking and identification of specific research activities and themes and explores patterns of the scientific network.

**Figure 1:**
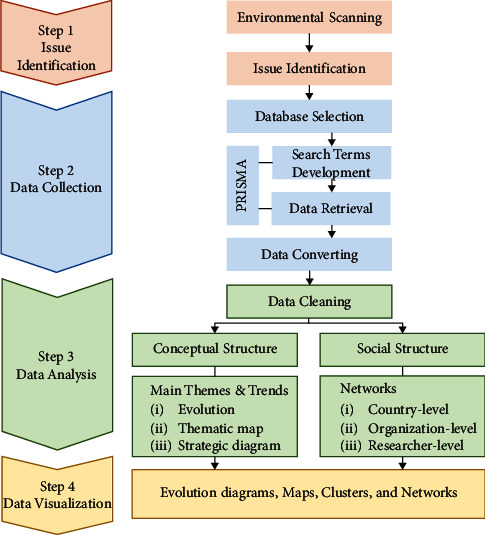
The process of scientometric analysis.

To achieve more comprehensive insights, we refined an approach developed in our previous research that combines traditional quantitative scientometric techniques with qualitative data derived from the evaluations of the shelf-life technology experts [24].  Figure 1 shows the overall research framework.

### 3.1. Data Collection

To identify search terms for collecting and retrieving scientific articles published from 2000–2020, we used Boolean search strings [25], combined with expert input to locate articles in the Web of Science (WoS) database. The year 2000 was chosen as the starting period based on an initial analysis showing that publications related to fruit shelf-life extension began increasing that year.

We used queries and searched for titles, abstracts, and keywords. The final search strategy was ‘TITLE-ABS-KEY (“shelf life” OR shelf-life OR “storage life”) AND (extension^*∗*^ OR extend^*∗*^ OR increase^*∗*^ OR improve^*∗*^ OR prolong^*∗*^ OR pro-long^*∗*^) AND (fruit^*∗*^) AND (postharvest^*∗*^ OR postharvest^*∗*^ OR fresh^*∗*^ OR “fresh cut” OR “fresh-cut”).

We employed the Preferred Reporting Items for Systematic Reviews and Meta-Analyses (PRISMA) guidelines to improve the reliability of our data collection process [26]. Following the prescribed steps, the total initial number of publications was 3,598 records. Targeted data sources included journal articles, editorials, books, book reviews, book chapters, and conference papers, whereas reprints and non-English documents were excluded. Eligibility screening also excluded articles with incomplete information (e.g., losses in lists of organizations and countries) and articles found to be irrelevant. Finally, a total of 3,131 documents were included in the scientometric analysis.

### 3.2. Data Analysis

We employed two scientometric software programs: VantagePoint version 13.1 (2020) (https://www.thevantagepoint.com/) and R-package Bibliometrix version 3.0 via Biblioshiny (https://www.bibliometrix.org/) to analyze the data.

Prior to conducting the analysis, data cleaning was done as a preprocessing step to remove errors and reduce duplication owing to variations in the names of authors, affiliations, and countries. Unmatched data were combined to facilitate standardization, and the “List Cleanup” tool—paired with the manual cleanup—was applied to unify the country, affiliation, and author names. Text manipulation algorithms were applied to those names and they also underwent careful manual inspection. Every affiliation and author name was converted into a term with the same meaning, which was also the case for terms such as “University,” “Uni,” and “U.”

For the data analysis, both softwares can conduct scientometric functions that help reveal relationships among authors and research fields as well as time series analysis and associated organizations, citations, and collaborations, among others [27]. Following the process shown in Figure 1, two main analyses, consisting of the conceptual structure analysis and the social structure analysis, are computed.

Specifically, for the conceptual structure analysis, we employed Bibliometrix to construct a strategic or thematic map and analyze the evolution. This analysis helps to understand the research trend in terms of publication and citation. Moreover, the research themes including research areas were analyzed by classifying those into main research fields. The evolution of research areas was also completed to construct the strategic diagram based on co-occurrence analysis, which aids in understanding the research density and centrality of each area [28]. To draw the strategic diagram, we used the keywords provided by the original authors rather than the commonly used KeyWord Plus, which extracts terms from article reference titles [29]. Although Keywords Plus is as effective as keywords by authors for conducting scientometric analyses of scientific and technical knowledge structures, Keywords Plus is more generally descriptive in article content rather than the representation of in-depth content in the articles [30]. The analysis procedure for constructing the strategic diagrams is summarized in the following.

Scientific maps called strategic or thematic diagrams have been proposed to visualize outputs from co-occurrence analysis to achieve strategic analysis [31]. The basic premise is that terms (e.g., keywords, terms, or phrases extracted from titles or abstracts) that frequently appear together in a publication are considered to be linked, and groups of such terms comprise a co-occurrence network. The strength of such relationships is expressed by the equivalency index (*e*_*ij*_) [32], defined in the following equation:

(1) $$\begin{array}{c} e_{ij}=\frac{C_{ij}^{2}}{C_{i}C_{j}}, \end{array}$$

where *C*_*ij*_ is the number of publications, in which the two keywords *i* and *j* co-occur, and *C*_*i*_ and *C*_*j*_, respectively, represent the number of publications in which each term is found. An equivalency index of one denotes that the keywords always appear together, whereas an index of zero means that they are never associated. Once the links have been quantified, an algorithm called “simple centers” makes two passes through the data to generate groupings or clusters of themes [32]. The first pass creates networks of internal links forming the strongest associations, whereas the second adds links of lesser strengths (i.e., external links) between networks. Once the analysis is conducted, it is possible to create a two-dimensional strategic or thematic diagram, in which themes are plotted along the *x*-axis according to Callon's centrality and the *y*-axis according to Callon's density [28, 32].

The density (*d*) denotes the network's level of internal cohesion and strength, as defined in the following equation:

(2) $$\begin{array}{c} d=100\times \frac{\sum^{}e_{ij}}{W}, \end{array}$$

where *i* and *j* are the keywords belonging to the theme and *w* is the number of keywords in the theme. *i* and *j* are the keywords related to the research theme, *w*is the total number of keywords in that research theme, and *e*_*ij*_ is the strength of keywords' relationships (the equivalency index). In other words, internal links represent the conceptual development of the theme.

Network centrality (*c*), i.e., the external cohesion index, measures the degree of thematic interaction with other networks, which is defined in the equation:

(3) $$\begin{array}{c} c=10\times \sum^{}e_{kh}, \end{array}$$

where *k* is a keyword associated with the research theme and *h* represents a keyword belonging to other research themes. Isolated networks have low centrality values.

In another point of analysis for the social structure analysis, both Bibliometrix and VantagePoint were applied. We analyzed a three-level collaboration network (e.g., country, affiliation, and individual level analysis). Descriptive and scientometric indicators to understand scientific and publication activities such as annual publication growth, research productivity, and collaboration index (CI) were used to achieve a data overview. In each level of analysis, the indicators such as research productivity and citation analysis were conducted. Furthermore, for the researcher level, a number of indices such as the *h*-index, *g*-index, publication behavior using Lotka's law, and collaboration index using single and multiple collaboration were also computed.

Specifically, the collaboration index (CI) adopted Lawani's concept to denote the average number of authors per article for a given set of articles [33]. This index is calculated as shown in equation (4) [34, 35]:

(4) $$\begin{array}{c} \text{CI}=\frac{\text{TA}u_{m}}{{\text{TA}}_{m}}, \end{array}$$

where TA*u*_*m*_ represents the total number of authors and TA_*m*_ is the total number of multiauthored articles.

### 3.3. Data Visualization

Following the analysis, we performed data visualization by creating graphical representations to demonstrate research evolution and analyze knowledge synthesis, namely, social structure, which reveals how actors interact with each other, and conceptual structure, which illuminates networks of keywords, citations, authors, institutions, and countries [36]. Generally, for the network and cluster involving the interaction among items, the circles represent the items (i.e., country, affiliation, etc.) under the analysis associated with the respective denomination. The connecting lines represent the related degree to which thicker lines refer to a higher connection. The concept of algorithms for the cluster network is summarized in the following.

Based on the concept of modularity (Q), this algorithm has a demonstrated ability to handle complex networks and shows high performance among popular algorithms [37, 38]. Modularity measures the quality of the network clusters detected by the algorithm, whereby larger values denote stronger community structures [38]. The Louvain algorithm consists of two steps. Each node is considered as belonging to a specific community, and adjacent nodes, whose merging results in higher modularity are combined in the same group. Small communities are identified by optimizing modularity on localized nodes, following which each community is treated as a node and the first step is repeated on the newly formed network [39].

After each community is identified, centrality measures are calculated to identify levels of strategic significance among nodes in the network [40]. There are mainly three indices to measure centrality: “degree centrality,” or the number of linkage lines between a node and others in a particular network, whereby more relational ties indicate greater prestige or influence; “betweenness centrality,” which denote a node's position between other nodes—nodes on the shortest path between other nodes are deemed the most “central” and control information flows between networks; and “closeness centrality,” or the degree, to which a particular node is close to all other nodes in the network.

## 4. Results

### 4.1. The Conceptual Structure Analysis

#### 4.1.1. The Evolution over Time

The publication and citation trends from 2000 to 2020 are shown in Table 1. The number of publications grew at an average compound annual growth rate (CAGR) of 17.1%, increasing from only 18 articles in 2000 to 424 articles in 2020. The first decade shows little progress, followed by a more prolific second decade. Typical explanations for such growth are that the overall number of researchers substantially increased over time. It is reflected in the number of journal submissions, and the rapid discovery of emerging trends in research fields [41].

At the time of our data collection, the year with the highest number of citations was 2011, when 4,242 citations were recorded, whereas 2000 was the most academically impactful year with 67 citations per publication. On average, the number of citations per publication decreased over time. This is likely due to the positive relationship between the length of time since article publication and the number of citations; earlier articles have obviously had more time to be incorporated in subsequent studies [42].

#### 4.1.2. Strategic Diagrams

To complement the scientometrics, co-occurrence analysis was conducted to identify the strategic relationships. This analysis was mapped to help visualize the division of the larger field of shelf-life technology into several themes or subfields and discern their relationships, thereby providing insights into the evolution of major research foci [11].

**Figure 2:**
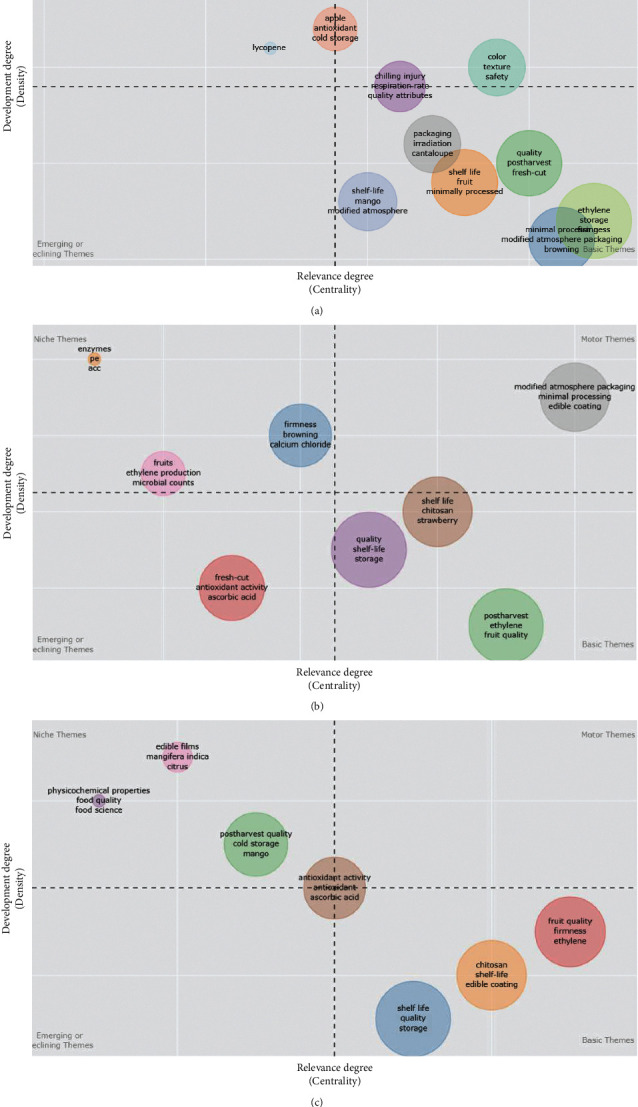
The strategic diagrams for shelf-life extension technology-related research from (a) 2000–2006, (b) 2007–2013, and (c) 2014–2020.

We considered three 7-year periods from 2000–2020 to understand the evolution of research interests. For each subperiod, authors' keywords were normalized to avoid trivial duplications, and a minimum threshold of three occurrences was set to filter out infrequent keywords. On each subperiod co-occurrence matrix, the multilevel algorithm based on a community detection procedure was performed [36]. We noted that each theme contains a number of keywords; however, the strategic evolution map in Figure 2 shows only the three keywords with the highest number of co-occurrences, which are the representations of major research interests in the themes. Each cluster or research theme is labeled with corresponding keywords, and cluster sizes are proportional to the number of word occurrences.

Themes in quadrant I (upper right) are considered the “motor themes” that present both high centrality and high density, meaning that they are well-developed and important for the structuring of a research field. The themes in this quadrant are also closely related to concepts belonging to other themes. Themes in quadrant II (upper left) are “very specialized themes” characterized by well-developed internal links (i.e., high density) but weak external interconnections (low centrality) that are of limited or marginal importance to the technological field. Themes in quadrant III (lower left) represent “emerging or disappearing themes” with limited centrality and low density, denoting that they are weakly developed and marginal—although the former may gain greater centrality over time as the topic gains traction. Themes in quadrant IV (lower-right) are considered “general basic themes” with strong centrality but less density. These transversal and basic themes are important but are either not developed or have limited internal development.

Eleven main research topics related to shelf-life extension technology emerged in the first subperiod (2000–2006; Figure 2(a)). We observed different terminology to describe the research domain. The strategic diagram indicated that the motor theme was “color,” which includes “texture” and “storage temperature,” and associated studies investigated the effect of antibrowning agents and storage time on the color of apples and color changes in grapefruit juice during cold storage [43, 44]. There are a number of essential research themes in the lower-right quadrant; however, there is limited developmental progress. The two most extreme themes in this quadrant are “ethylene” and “minimal processing.” On the left, there are neither declining nor emerging research areas in the first 7-year period. One niche theme of this period was “apples.” Under this cluster, studies include those investigating the effect of “gamma radiation” to defer ripening and increase shelf-life; however, this subtechnology has limited global activity and shows no further development during later periods [45].

In the second subperiod (Figure 2(b)), the research areas have merged into eight groups. In particular, a “modified atmospheric packaging” cluster has moved from being a basic theme during the first subperiod to becoming a prominent motor theme with extensive development. Unlike ethylene, this subtechnology is a nonchemical treatment, and its increasing prominence indicates a trend toward maintaining food safety with minimal environmental cost [46]. Interestingly, “antioxidant activity” (one of the main quality indices affecting fruit shelf-life) was observed to be a declining theme—both centrality and density values decreased from the previous subperiod. Other basic themes of this period were “storage” and “ethylene,” whereas “enzyme,” “microbial count,” and “firmness” emerged as specialized themes in quadrant II. Notably, “poverty/children” began to move from the periphery to a more central position with a high density and medium centrality level.

We observed a different topical distribution during the final subperiod (Figure 2(c)). There is no evident research theme to be classified as motor themes during the most recent decade, as both chemical and nonchemical treatment have already been developed, and some subtechnologies have already been utilized in commercial applications. Similarly, we observed no emerging or declining themes. However, “physicochemical properties” (e.g., texture) became a more specialized theme, indicating that a number of research teams or groups have focused on this topic. Apart from the goal to extend storage life, researchers also concentrated on the effect of these properties on taste, which heavily influences purchasing decisions. Interestingly, an “antioxidant activity” research group has moved to the central of the strategic diagram, which denotes that the group's future activity depends on research interests and policies from leading countries. The “storage” and “ethylene” groups remain among the general themes; however, “chitosan” (a natural substance for coating and preserving fruits) has moved to the basic themes, decreasing in density along with evincing a slight increase in centrality, meaning that research development has achieved a certain level of importance.

### 4.2. The Social Network Analysis

#### 4.2.1. Country-Level Networks and Scientific Outputs

Table 2 shows the macro, country-level analysis of the ten most active countries according to numbers of publications and citations. In this analysis, the countries of corresponding authors were considered.

China had the highest total number of articles, very distantly followed by India and Spain. With a population of approximately 1.4 billion, China has invested vast resources and promulgated policies to ensure the security of food supplies during recent decades [47], and local governments are enjoined to implement policies to support scientific and technological development [48]. India and Spain are rich in biodiversity-related to fruit and vegetables. Notably, Spain, a relatively tiny country, is the leader for total citations followed by China and the U.S. If one considers both total articles and citations, the U.S. and Spain have been the most productive and influential. Spain reported the highest average citations per article with 39 citations. Although the U.S. contributed only 7.5% of total publications, it receives nearly 34 citations per article (with 13.2% of total citations). China has produced more publications and total citations but earned fewer citations per article because of large proportion of total China's articles.

The investigation of research collaboration assists in strengthening the understanding of information and resources. For the collaboration among countries, co-authored publications across countries are frequently used to measure international collaboration.  Table 3 presents the top ten countries engaged in collaboration. This measurement was evinced by the number of studies published by authors from the same country (measured as single country articles, SCA) or different countries (measured as multiple countries articles, MCA). All of the top ten countries evince high SCA values relative to MCA, thereby indicating a low tendency toward cross-national collaboration. The cross-collaboration ratio (as computed by MCA/TA) ranged from low (less than 10%) to moderate (above 28%) values. Notably, Spain, the U.S., and Mexico exhibit high levels of cross-national collaboration (more than 25% of total articles), whereas India, the second-ranked country for SCA, shows the least cross-national collaboration.

**Figure 3:**
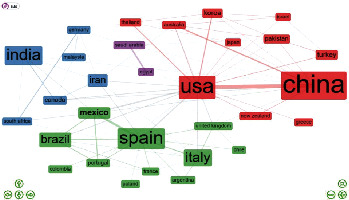
The research networks among the top 30 science-producing countries.

To visualize cross-national collaboration networks, we examined the top 30 countries with at least two co-authored publications. To explore groups of countries with similar collaboration patterns, an algorithm was performed by using Louvain community detection proposed by Blondel et al. [49]. In Figure 3, nodes denote countries, links represent co-authorships, and the size of nodes reflects the number of articles in each country. The thickness of the linkage line connecting two nodes (e.g., countries) is proportional to the strength of co-occurrence; heavier lines indicate a high degree of relationship between each pair of countries. A networking community is defined as a group of nodes that more frequently interact among themselves than with nodes from other groups.

Networks among the 30 leading countries are represented by a diagram (Figure 3) with four communities represented by red, blue, green, and purple nodes. The U.S. exhibits the highest betweenness centrality (176.16), followed by Spain (89.41) and China (39.74). These three countries control the flow of information. Apparently, the U.S. controls knowledge flows both within (red cluster) and across clusters. In terms of strength of collaboration, the U.S. mainly collaborates with China, whereas Mexico mainly collaborates with Spain.  Table 3 shows that Spain and Mexico each have similar cross-collaboration ratios of around 28%, which is consistent with the pattern shown in Figure 3. Most of the countries in the red and blue clusters are from a mixture of regions including North America, Asia, and Australia. Although the green cluster occupies the second tier in terms of the number of countries, it is primarily comprised of European countries, which reflects the close geopolitical connections in the region. In the purple cluster, both countries are next to and show moderate ties to each other.

**Figure 4:**
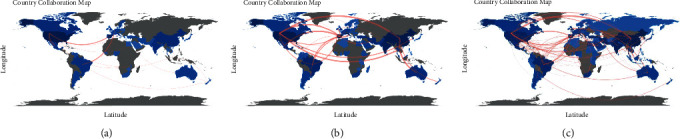
World map showing research collaborations among countries (a) from 2000 to 2006, (b) from 2007 to 2013, and (c) from 2014 to 2020. Brighter blue coloring indicates a higher collaboration rate.

To elaborate on global collaboration networks and understand the dynamics of collaboration over time, we generated a country collaboration map by dividing the 21-year period into three subperiods (2000–2006, 2007–2013, and 2014–2020) to identify changes over time (Figure 4). We again set a threshold of at least two co-authored publications. As indicated by the number of linkage lines, global collaboration networks have increased over time. The thickness of the linkage lines represents the number of times of collaboration; thicker lines denote more collaborations.

From 2000–2006, the most frequent collaborations were between the U.S.-Mexico (six times), the U.S.-Spain (three times), and Brazil-Netherlands (three times). From 2007–2013, the top collaborators shift to the U.S.-China (nine times), followed by China-Australia (eight times), Spain-Mexico (eight times), and the U.S.-Thailand (eight times). During the most recent period (2014–2020), the top collaborator remains the U.S.-China (39 times), followed by Egypt-Saudi Arabia (16 times), and Spain-Mexico (14 times). When looking at the overall time period, the results reflect the results aligned with Figure 3, as the U.S.-China remains the top collaborator (49 times), followed by Spain-Mexico (22 times), and Egypt-Saudi Arabia (18 times). Notably, the rate of collaboration between the U.S. and China has substantially increased compared with other countries. The first two groups work across regions, whereas Egypt and Saudi Arabia are both in the Middle East and North Africa (MENA) region and face similar challenges in postharvest management [50].

#### 4.2.2. Organization-Level Networks and Scientific Outputs

According to the publications obtained from the database, 1,992 organizations have conducted research on shelf-life extension technology. We classified organizations into four groups: academic institutions (e.g., universities, 77.2%), government entities and their research institutes (16.2%), corporations (5.7%), and other types of organizations such as hospitals and individual researchers (0.9%).

We investigated the 30 organizations with the most publications by exploring their dominant research themes and areas of focus. After obtaining research categories from the Science Citation Index Expanded (SCIE) database, we consulted with technical experts to obtain a concise definition of each category, and then we summarized the definition of the top three categories (Table 4). Relationships were mapped by identifying linkages among the organizations (represented as nodes) according to the values on the list delineating the top three research themes, which are food science and technology, horticulture, and agronomy.

**Figure 5:**
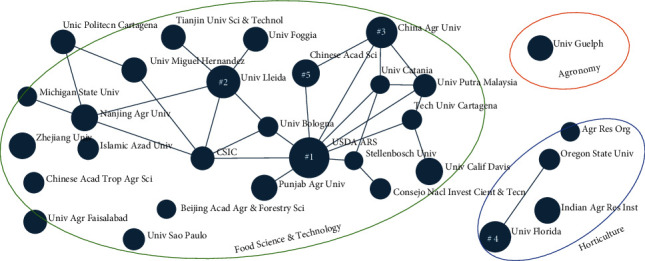
The cross-correlation map of the top 30 science-producing organizations and associates research themes (nodes numbered one through five represent the five organizations with the most publications in descending order).

The cross-correlation map shown in Figure 5 shows relationships among the top 30 organizations and the research areas, on which they are most focused. The relationships indicate most organizations that have been working on similar research areas. Each organization has performed research in most of the research areas; however, the groupings indicate their strongest foci. The most productive organization is the U.S. Department of Agriculture-Agricultural Research Service (USDA-ARS) with 93 publications, followed by the University of Lleida in Spain (61 publications), China Agricultural University (57 publications), the University of Florida in the U.S. (57 publications), and the Chinese Academy of Sciences (44 publications). Although India is the second-ranked country in terms of the total number of articles, that country is not represented among the top five organizations.Whereas the University of Florida emphasizes horticulture, the other top four organizations concentrate on food science and technology. Notably, the first-ranked organization is the government entity. The USDA-ARS has established a “Global Food Security Strategy” and collaborates with all levels of government agencies, universities, and the private sector at home and abroad to tackle critical agricultural challenges [51]. All of the analyzed categories except agronomy are connected to a group of countries linked by solid lines. Notably, none of the top five organizations are prominently engaged in agronomy and only one of them substantially focuses on horticulture. Thus, future research can prioritize technological development in these areas.

#### 4.2.3. Author-Level Networks and Scientific Outputs

Table 5 provides that the articles were written by 9,095 researchers, with an average value of 0.344 publications per author. Only 0.5% of these articles were produced by a single author; the average number of authors per article is about 2.90 (the inverse of 0.344 or the number of authors divided by the number of articles). The number of co-author per document (number of author appearances divided by the number of articles) is about 4.68. The difference between these two indexes is due to the different methods to count authors [27]. The number of authors publishing single or co-authored articles can serve as a proxy for the average size of the researcher groups or teams; however, publications with large numbers of co-authors can substantially increase this indicator [36].

Due to the complexity of interactions among authors, it can be challenging to determine the precise magnitude and nature of collaboration over time. Thus, we applied the collaboration index (CI) as an indicator for measuring the collaborative work among researchers. As Table 5 provides, the CI ratio is roughly 2.96. In other words, on average, approximately three authors were involved in the production of each article regardless of national or international collaboration. The CI ratio (2.96) is close to the author per document ratio (2.90) due to the very small number of single-authors articles, compared to the number of multiple-authored articles.

Lotka's law defines an inverse relationship between the number of articles and the number of researchers authoring the publications. Lotka's law affirms that the number of authors producing publications decreases as the number of published articles increases [52]. Equation (5) illustrates Lotka's law as follows:

(5) $$\begin{array}{c} A_{n}=\frac{A_{1}}{n^{2}}, \end{array}$$

where *A*_*n*_ represents the number of authors, who published *n* articles, *A*_1_ represents the number of authors, who published one article, and *n* represents the number of articles (*n* = 1, 2, 3, ..., *k*, where *k* is the maximum number of published articles of an author). Lotka found that the number of authors, who published *n* articles is about 1*/n*^2^ of the number of authors, who write one article (*A*_1_) [52]. In other words, authors, who write one article account for a percentage of about 60%. Thus, we assumed that the exponential term to calculate the power value is unknown. If we set the variable at *x*, then the equation would be *A*_*n*_=(*A*_1_/*n*^*x*^).

**Figure 6:**
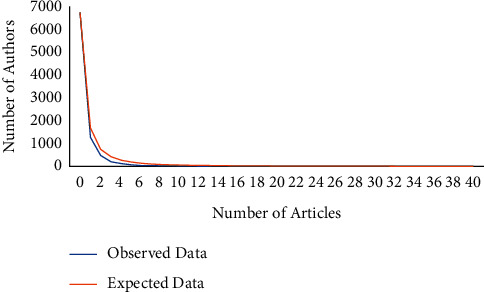
The frequency distribution of scientific production.

The value of exponent *x* in the field of shelf-life extension technology research is approximately 2.59 (higher than 2), which indicates that Lotka's law overestimates the number of authors in this field. To more clearly display the distribution difference, the plot of Lotka's law is shown in Figure 6. According to our data analysis, authors, who have published only one article during the 21-year period account for 74.4% of the total, whereas authors, who have published at least five articles account for only 3.9%. It can be interpreted that the core authors of this technology have published many articles. The highest number of articles was 39 publications with one author (Table 6). This phenomenon can be explained by noting that the distribution of observed value is highly skewed to the right. This skewness demonstrates that the publication numbers of prolific authors in this field are below the expected level [53]. In other words, this field is not yet a mature field and is still in a stage of continuous development.

Table 6 presents the ten researchers responsible for the greatest contributions to shelf-life extension technology research along with total articles and total citations during the period. O. Martín–Belloso at the University of Lleida in Spain (the second-ranked organization) was the most productive author. In other cases, the researcher's level of production is less closely correlated with the rank of their organization. For example, M. Serrano and D. Valero at University Miguel Hernández in Spain (12th ranked organization) were the second and the third highest contributors, respectively. Overall, the top ten researchers are from Spain, China, and India, which are the three countries that contributed the most publications.

The main research category of each author was mapped using a cross-correlation map between author names and research themes with a similar algorithm to that described in the previous section. We found that nine of the ten researchers mainly focus on food science and technology research areas, whereas only one concentrates on horticulture. Notably, no researcher from the first-ranked organization, USDA-ARS, was observed among the top ten researchers; however, as a government agency, it seems likely that it supports research grants and funding to universities and industries [51].

In addition, Table 6 highlights O. Martín–Belloso as the most influential author with 2,785 total citations and 71.4 citations per article, followed by M. Serrano. Overall, most researchers from Spain have accomplished a large number of total citations and total citations per article, indicating that they have produced high-impact research works that are useful for citation by other researchers worldwide.

We were unable to specify if citations were self-citations or even positive/negative citations; however, the *h*-index is purported to perform better than other indicators used to evaluate authors' scientific output such as the total number of documents and the total number of citations [54, 55]. The *h*-index denotes the number of an author's papers that have been cited at least (*h*) times. Moreover, we also obtained each author's *g*-index, a measure of the global citation performance that was introduced to overcome the limitations of the *h*-index [56]. Our analysis found some alignment between these indices and the numbers of publications and citations among the top ten researchers. O. Martín–Belloso was again identified as the author with the highest indices, with an *h*-index of 29 and a *g*-index of 39, followed by M. Serrano and D. Valero, each of whom obtained an *h*-index of 26 and a *g*-index of 33.

**Figure 7:**
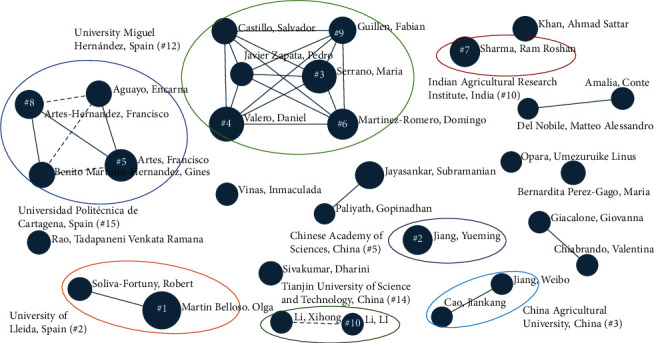
Auto-correlation map of the top 30 researchers and their organizations (nodes numbered one through ten represent the ten researchers with the most publications in descending order).

Last, we prepared an autocorrelation map to conduct relationships among the top 30 researchers and organization groups.  Figure 7 shows strong (thick solid lines), moderate (thin solid lines), weak (dashed lines), and no relationships (no line) between researchers. Overall, the top 30 researchers have primarily worked within their organizations. For example, O. Martín–Belloso has largely conducted research with colleagues at the University of Lleida. Similarly, researchers associated with the two large clusters represented by University Miguel Hernández and Universidad Politécnica de Cartagena have worked together to produce scientific outputs. University Miguel Hernández evinces the strongest linkage lines, whereas moderate and weak relationships were observed for Universidad Politécnica de Cartagena. This indicates that they may publish works with others not listed among the top 30 researchers shown in Figure 7. Similarly, other organizations show quite small clusters or groups of research teams; however, their cross-collaboration ratios indicate that they regularly collaborate with researchers at other organizations.

Whereas relationships across organizations are rarely observed, the collaboration map is based on the number of co-occurrences whereby researchers have copublished. Therefore, the impact of the number of co-publications within one organization is stronger than that of co-publications across organizations. However, as shown in Figure 5, many organizations collaborate both domestically and internationally; thus, large networks can be further partitioned into collaboration groups based on either formal or informal linkages. Moreover, rather than remain limited to linear collaborations, organizations can form centralized networks, in which a single (dominant) organization connects many others across multiple research themes, thereby resulting in more outputs and less time to market.

## 5. Discussion and Implications

The results presented above can play an important role in tracking both the development of and challenges facing public health and social policy. Communication is the key to forming connections that link socially-driven technologies capable of aiding countries and promoting industry. By pursuing partnerships with universities and research institutes, firms can reduce development time via knowledge-based resources [12]. While the industry's primary concern is the technological application in a business context, academics prioritize the underlying science and its various impacts. The issue of extending food shelf-life unites these two ambitions, enabling the two sides to work in harmony toward the same goal. Two effective means, by which to achieve this are to support corporate funding for university research and to engage in knowledge, idea, and scholar exchange programs [57]. The results can also inform policymakers regarding initiatives to support national development, including those that fund area of research that may be less popular despite their importance regarding economic competitiveness and social sustainability moving forward.

The recent evolution of research into both the bioeconomy (BE) and the circular economy (CE) was provided by Abad–Segura et al. [58], who went on to analyze the implications of joint implementation on sustainability. It is clear that environmental policies are essential, and the multidisciplinary nature of the research field may be driving the expansion of scientific development [59]. In fact, a multidisciplinary approach can help to identify the various areas, in which policy decisions must favor sustainability. Four key areas of focus regarding BE were identified by Bröring et al. [59]: substitute products, bio-based processes, bio-based products, and new consumer behaviors. It is essential to maintain a balance in resources and raw materials to ensure sustainability, not just in the products themselves [60]. New technologies designed to extend food shelf-life need to take these issues into account to aid innovation.

A recent review of advanced bio-based packaging material technologies by Soro et al. [61] found that biodegradable raw materials, such as polysaccharide-based film, can enhance food shelf-life due to their physical properties and nontoxic substances [62]. This finding, which is consistent with our results regarding firms working in niches, shows how waste from spoiled food can be reduced in the agri-food sector. This will encourage and attract future research.

This study highlighted the importance of engaging in cross-collaboration to produce high-impact works. The exchange of knowledge and ideas from different regions could lead to substantial innovations in R&D. Global collaboration networks enable researchers to combine their unique knowledge, experience, and resources to develop and transfer solutions to shared agricultural challenges. However, researchers may need to conduct a cost-benefit analysis to ensure that the benefits conferred by such partnerships outweigh the cost to their individual prominence [63]. We propose that the urgent need for shelf-life technology to reduce food losses and waste presents an incentive for resource- and knowledge-exchange toward innovative and positive outcomes. Our analysis demonstrates that many institutions have developed specialized research processes and foci. Related stakeholders can benefit from such analyses by using them to create profiles of organizational research patterns. The collaborative networks assist in consolidating resources and areas of specialization or research additional topics to address national or regional needs. In the context of the technology for extending shelf-life, Spain, China, and the U.S. serve as primary hubs for knowledge exchange.

## 6. Conclusions

Technological disruption has been the hallmark of the fourth industrial evolution, making businesses increasingly eager to seek out new strategies regarding technological growth. To succeed in such a competitive landscape, companies need to forecast emerging technological trends and adjust their market positions accordingly. Data obtained from technological databases such as scientific articles, is one way to gain valuable insights into how firms and governments should go about planning their futures.

This study presented an application of a scientometric analysis to analyze the conceptual structure of knowledge and social networks to address the research questions using shelf-life extension technology for food as a case study. Maps of research clusters were generated to illustrate the usefulness of the results for decision-making. For the conceptual analysis, we found that specialized themes indicated by an increase in centrality can be considered as potential subjects for intensified attention and expansion to other researchers. The trend toward bio-based materials, which involves a joint application of concepts in the circular economy and bioeconomy, is particularly interesting. Policy-driven governance is the method, by which to generate more accurate research development including regulatory frameworks and food safety standards.

For the social analysis, we found that cross-collaboration ratios among the top 10 countries engaged in food preservation research vary from less than 10% up to 30%. We identified strong cross-national networks linking the U.S. and China, Spain and Mexico, the European Union, and Egypt and Saudi Arabia. At the organizational and individual levels, we found that most organizations and researchers have focused on food science and technology research versus horticulture or agronomy; hence, the latter fields can be further explored to initiate novel scientific contributions to this technology.

Our previous research explored that the leading entities in this technology in terms of technology development (patents) are universities [64]. In the present work, we also observed that universities are the top players in terms of scientific development (articles). Specifically, we found the same top three players in both articles and patents, which are China Agricultural University, Zhejiang University, and Nanjing Agricultural University. Hence, this technology is led by universities to promote and support food sustainability.

The results demonstrate the advantages of this approach for analyzing raw big data to understand R&D activity and productivity through scientometric indices. In particular, they highlight the utility of using a mixed-method approach to trace the evolution of research themes, frameworks, and networks, providing essential inputs for further decision-making. Researchers, practitioners, and policymakers can utilize these resources to develop guidelines for technology opportunity analysis, as well as to discover and identify emerging or underdeveloped areas for future research. In addition, the findings indicate the potential of this process for identifying collaboration opportunities between different parties.

## 7. Limitations and Future Research

This research contributes to efforts to understand scientific activities and research networks by illuminating research areas and key players related to the shelf-life extension technology. However, this study has a few limitations that can be addressed in future research.

First, we limited our article search to the Web of Science database. Other peer-reviewed publications and non-peer-reviewed sources may be useful. No perfect database exists, and deriving sources from different databases may lead to varying results. Thus, articles can be extracted from other databases to analyze and compare findings.

Second, the present study was limited to the English language, and the source categories were restricted. Some research papers in other categories and in other languages may have been overlooked. For instance, patents, an important source of information on R&D, were not included in our analysis. Future studies could consider expanding the scope of languages and document types.

Finally, various representatives from universities, research institutes, and industry can also be invited to interpret the results. Food preservation involves multiple disciplines, including chemistry, food science, physics, and engineering, among others. Workshops or focus groups can elaborate on the implementation and utilization of policies or other measures to achieve common goals and map future research directions.

Based on the findings from the conceptual structure analysis, future researchers can explore the emerging topics or fields of this technology to understand trends and priorities for further R&D. Furthermore, researchers can also utilize insights from the findings to create a product roadmap or portfolio. The new product development (NPD) process can be formed effectively because of the insights generated regarding emerging and declining themes.

The findings presented in this research will also aid policymakers in developing more effective policies regarding technology roadmaps and collaboration. Future technological advancements will lead to increasingly collaborative approaches, with research organizations and universities at the heart of such processes. These groups will be among the main benefactors of technological improvements, enabling researchers to use the findings to generate advanced insights into the commercialization of such technologies and to examine their wider applications.

Considering the above-stated limitations, the publications analyzed in this study might not reflect the entire scope of global research activity related to fruit shelf-life; however, the data, we have presented provide substantial insights that can be used to inform further investigation into this increasingly important issue.

## Figures and Tables

**Table 1:** The publication and citation trends.

Year	TA	CTA	TC	TCA
2000	18	18	1,206	67.00
2001	18	36	1,138	63.22
2002	32	68	1,759	54.97
2003	49	117	2,362	48.20
2004	42	159	2,119	50.45
2005	61	220	2,920	47.87
2006	58	278	2,334	40.24
2007	93	371	3,709	39.88
2008	79	450	3,708	46.94
2009	102	552	3,633	35.62
2010	116	668	3,622	31.22
2011	129	797	4,242	32.88
2012	116	913	2,778	23.95
2013	153	1,066	4,048	26.46
2014	166	1,232	3,790	22.83
2015	233	1,465	4,491	19.27
2016	229	1,694	3,104	13.55
2017	258	1,952	3,223	12.49
2018	335	2,287	2,733	8.16
2019	420	2,707	2,316	5.51
2020	424	3,131	823	1.94

TA, total articles; CTA, cumulative total articles; TC, total citations; TCA, total citations per article.

**Table 2:** Top 10 scientific production countries based on articles and citations.

No.	Country	TA	% of share	No.	Country	TC	% of shares	TCA
1	China	530	17.0	1	Spain	10,414	17.4	39.2
2	India	296	9.5	2	China	8,786	14.7	16.6
3	Spain	266	8.5	3	The U.S.	7,911	13.2	33.7
4	The U.S.	235	7.5	4	Italy	4,455	7.4	20.6
5	Italy	216	6.9	5	India	3,176	5.3	10.7
6	Brazil	175	5.6	6	Brazil	2,314	3.9	13.2
7	Iran	140	4.5	7	Portugal	1,726	2.9	30.3
8	Turkey	94	3.0	8	Iran	1,658	2.8	11.8
9	Korea	88	2.8	9	Mexico	1,555	2.6	19.0
10	Mexico	82	2.6	10	Canada	1,332	2.2	26.1

TA, total articles; TC, total citations; TCA, total citations per article.

**Table 3:** The collaboration level of the top ten scientific production countries.

No.	Country	TA	SCA	MCA	Cross-collaboration ratio (%)
1	China	530	443	87	16.4
2	India	296	276	20	6.8
3	Spain	266	191	75	28.2
4	The U.S.	235	174	61	26.0
5	Italy	216	188	28	13.0
6	Brazil	175	141	34	19.4
7	Iran	140	124	16	11.4
8	Turkey	94	87	7	7.4
9	Korea	88	70	18	20.5
10	Mexico	82	59	23	28.0

TA, total articles; SCA, single country articles; MCA, multiple country articles.

**Table 4:** The categories based on Web of Science (WOS) and expert determinations.

No.	Category name	Definition
1	Food science and technology	“Resources concerning various aspects of food research and production, including food additives and contaminants, food chemistry and biochemistry, meat science, food microbiology and technology, dairy science, food engineering and processing, cereal science, brewing, and food quality and safety.”
2	Horticulture	“Resources concerning the cultivation of flowers, fruits, vegetables or ornamental plants, in gardens, orchards, or nurseries.”
3	Agronomy	“Resources on the selection, breeding, management, and postharvest treatment of crops including crop protection and science, seed science, plant nutrition, plant and soil science, soil management and tillage, weed science, agroforestry, agroclimatology, and agricultural water management.”

**Table 5:** Analysis of co-authorship from 2000 to 2020.

Issue	Description	Results
Author	Authors	9,095
	Author appearances	14,666
	Authors of single-authored documents	48
	Authors of multiauthored documents	9,047

Documents	Documents	3,131
	Single-authored documents	60
	Multiauthored documents	3,071

Author–Document	Documents per author	0.344
	Authors per document	2.90
	Co-authors per document	4.68
	Collaboration index (CI)	2.96

**Table 6:** The profile of the top 10 researchers.

No.	Author	Organization	TA	TC	TCA	*h*-index	*g*-index
1	Martín–Belloso O.	University of Lleida, Spain	39	2785	71.4	29	39
2	Yueming J.	Chinese Academy of Sciences, China	34	1155	34.0	17	33
3	Serrano M.	University Miguel Hernández, Spain	33	2100	63.6	26	33
4	Valero D.	University Miguel Hernández, Spain	33	2084	63.2	26	33
5	Artés F.	Universidad Politécnica de Cartagena, Spain	30	972	32.4	16	30
6	Martínez–Romero D.	University Miguel Hernández, Spain	27	1844	68.3	24	27
7	Roshan Sharma R.	Indian Agricultural Research Institute, India	26	185	7.1	7	12
8	Artés–Hernandez F.	Universidad Politécnica de Cartagena, Spain	24	391	16.3	11	19
9	Guillen F.	University Miguel Hernández, Spain	24	1647	68.6	21	24
10	Li L.	Zhejiang University, China	22	295	13.4	11	16

TA, total articles; TC, total citations; TCA, total citations per article.

## Data Availability

The data used to support the findings of this study are available from the corresponding author upon request.
